# Cross-cultural adaptation and psychometric properties of the Brazilian-Portuguese version of the VSP-A (Vécu et Santé Perçue de l'Adolescent), a health-related quality of life (HRQoL) instrument for adolescents, in a healthy Brazilian population

**DOI:** 10.1186/1471-2431-11-8

**Published:** 2011-01-27

**Authors:** Mariana T Aires, Pascal Auquier, Stephane Robitail, Guilherme L Werneck, Marie-Claude Simeoni

**Affiliations:** 1 Service de Santé Publique - EA3279, Faculté de Médecine, Université de La Méditerranée, Marseille, France; 2 Instituto de Medicina Social, Universidade do Estado do Rio de Janeiro, Rio de Janeiro, Brasil; 3 Instituto de Puericultura e Pediatria Martagão Gesteira, Universidade Federal do Rio de Janeiro, Rio de Janeiro, Brasil

## Abstract

**Background:**

Health-related quality of life (HRQoL) assessment, encompassing the adolescents' perceptions of their mental, physical, and social health and well-being is increasingly considered an important outcome to be used to identify population health needs and to provide targeted medical care. Although validated instruments are essential for accurately assessing HRQoL outcomes, there are few cross-culturally adapted tools for use in Brazil, and none designed exclusively for use among adolescents. The Vécu et Santé Perçue de l'Adolescent (VSP-A) is a generic, multidimensional self-reported instrument originally developed and validated in France that evaluates HRQoL of ill and healthy adolescents.

**Purpose:**

To cross-culturally adapt and validate the Brazilian-Portuguese version of the VSP-A, a generic HRQoL measure for adolescents originally developed in France.

**Methods:**

The VSP-A was translated following a well-validated forward-backward process leading to the Brazilian version. The psychometric evaluation was conducted in a sample of 446 adolescents (14-18 years) attending 2 public high schools of São Gonçalo City. The adolescents self-reported the Brazilian VSP-A, the validated Psychosomatic Symptom Checklist and socio-demographic information. A retest evaluation was carried out on a sub-sample (n = 195) at a two-week interval.

The internal construct validity was assessed through confirmatory factor analysis (CFA), multi-trait scaling analyses, Rasch analysis evaluating unidimensionality of each scale and Cronbach's alpha coefficients. The reproducibility was evaluated by intra-class correlation coefficients (ICC). Zumbo's ordinal logistic regression analysis was used to detect differential item functioning (DIF) between the Brazilian and the French items. External construct validity was investigated testing expected differences between groups using one-way analysis of variance (ANOVA), Mann-Whitney tests and the univariate general regression linear model.

**Results:**

CFA showed an acceptable fit (RMSEA=0.05; CFI=0.93); 94% of scaling success was found for item-internal consistency and 98% for item discriminant validity. The items showed good fit to the Rasch model except 3 items with an INFIT at the upper threshold. Cronbach's Alpha ranged from 0.60 to 0.85. Test-retest reliability was moderate to good (ICC=0.55-0.82). DIF was evidenced in 4 out of 36 items. Expected patterns of differences were confirmed with significantly lower physical, psychological well being and vitality reported by symptomatic adolescents.

**Conclusions:**

Although DIF in few items and responsiveness must be further explored, the Brazilian version of VSP-A demonstrated an acceptable validity and reliability in adolescents attending school and might serve as a starting point for more specific clinical investigations.

## Background

Adolescents currently comprise 20% of the world's population and 85% of them live in developing countries [1]. In Brazil, there are about 35 million adolescents aged 10-19 years, representing 21% of the country's population [2]. The vulnerability of this large age group highlights the need for community health evaluation and medical care in this population.

The World Health Organization (WHO) defines adolescence as a period of "transition from childhood to adulthood, during which young people experience changes following puberty, but do not immediately assume the roles, privileges and responsibilities of adulthood" [3]. Adolescents undergo rapid biological, psychological and social developmental changes that result in diverse vulnerabilities. Social transitions and health progress have changed adolescent health; today their health problems are often related to risky behaviour and chronic illness. In developing countries, violence and accidents are the main determinants of morbidity and mortality in this age group [4]. In these countries, adolescents also face problems such as early pregnancy, school drop-out and substance abuse [4-6].

Therefore, health-related quality of life (HRQoL) measures, assessing adolescents'

physical, emotional, and social health and well-being, is increasingly considered an important outcome to be used to identify population health needs and to provide targeted medical care. Although in the last decades the HRQoL of adolescents has been investigated in many European, American and Asian countries [7-9], data are lacking on the general aspects of HRQoL for Brazilian teens.

In order to better examine Brazilian adolescents' HRQoL, appropriate measures with sound psychometric properties studied in this specific context of use are needed. The WHO's definition of HRQoL emphasised that quality of life is largely dependent on culture and values on health perceptions, physical well-being, social roles and cognitive functioning [10,11]. This has several implications. First, instruments to be used in adolescence are to be based on a HRQoL definition relevant to this age-group and thus should be developed according to the teen's point of view and account for their maturity and cognitive development. Second, whenever possible, the questionnaires should be self-reported by the adolescents in order to elicit the adolescents' perception on their own quality of life [12-15]. Third, a special attention should be paid to respecting cultural differences in the appraisal of the HRQoL of the individuals.

Few instruments evaluating teens' HRQoL have been validated for use in the Brazilian socio-cultural context. Two instruments are condition-specific HRQL instruments. The Child Perceptions Questionnaire (CPQ11-14) measures the impact of oral health abnormalities on the quality of life of children [16,17], whereas the Childhood Health Assessment Questionnaire (CHAQ) assessing functional ability in daily living activities initially was developed to be used in children and adolescents with juvenile idiopathic arthritis but also applied in other disabling conditions [18,19]. Two others are generic HRQL instruments.

The Child Health Questionnaire (CHQ) [18,20] was modeled after the SF-36 to survey health status in the Medical Outcomes Study. The child self-report version of the CHQ consists of 87 items, and was developed for children from 10 years of age; a version that can be completed by the parent is available in 2 lengths - 50 or 28 items [21,22]. It comprises 14 constructs: physical functioning, role/social functioning, general health perceptions, bodily pain, parental time impact, parental emotional impact, role/social behavioural, role/emotional behavioural, self-esteem, mental health, general behaviour, family activities, family cohesion and change in health. Cronbach's alpha ranged from 0.43 to 0.97 for all the scales [21].

The Pediatric Quality of Life Inventory (PedsQL) [8,23-27] was derived from the Pediatric Cancer Quality of Life Inventory (PCQL), and underwent several refinements to originate the generic measure [24]. The PedsQL 4.0 Generic Core Scales is a brief and easy to score instrument that can be administrated through a child self-report and a parent proxy-report to assess quality of life of children and adolescents with ages ranging from 2 to 18 years [8]. The instrument encompasses the following constructs: physical functioning (8 items), emotional functioning (5 items), social functioning (5 items) and school functioning (5 items) [8,23,24]. The item-scale correlations of the 23 items of the PedsQL showed that most items (19/23) for self-report and all items for proxy-report met or exceeded 0.40 [8]. Most self-report scales and proxy-report scales of the PedsQL approached or exceeded the minimum reliability standard of 0.70. When chronically ill, acutely ill, and healthy children were compared using the PedsQL, the scales demonstrated differences among the three groups, that is, healthy children presented higher scores than acutely or chronically ill children in all constructs [8].

The Vécu et Santé Perçue de l'Adolescent (VSP-A), developed in France, evaluates the HRQoL of ill and healthy adolescents. It is a generic, multidimensional self-reported instrument whose items were generated from individual interviews and focus groups conducted with adolescents [28,29]. It was specifically designed for this age group and has been validated in other countries including Spain and Colombia [30,31]. The VSP-A comprises 36 items assessing the following constructs: psychological well-being (5 items), physical well-being (4 items), body image (2 items), vitality (5 items), relationship with friends (5 items), relationship with parents (4 items), relationship with teachers (3 items), sentimental and sexual life (2 items), leisure activities (4 items) and school performance (2 items) [28-34]. All the VSP-A scales achieved a Cronbach alpha of at least 0.74 [28]. Item-scale correlations of the VSP-A demonstrated that each item achieved the 0.40 standard for item-convergent validity; the correlation of each item with its constitutive scale was higher than with the others [28]. The original VSP-A was applied to healthy adolescents, a group of adolescents presenting with an acute condition and a group of adolescents with a chronic disease. Healthy adolescents reported a significantly higher HRQL than others on most of the contructs [28,29].

The psychometric properties of PedsQL, CHQ and VSP-A are similar but there are some differences in the constructs assessed. Four aspects were considered when selecting the VSP-A to be cross-culturally adapted for use among Brazilian adolescents. First, VSP-A showed to be a robust instrument, that is easy to complete and score. Second, compared to the PedsQL and CHQ, VSP-A is the solely instrument designed exclusively for use among adolescents and developed according to the teens' point of view, which is fundamental for a HRQoL instrument, as this concept relies on the individual's perception. Third, it comprises fundamental constructs regarding the teen's HRQoL, including relationship with parents, friends and teachers, as well as body image and sentimental and sexual life. Nowadays, those concepts are recognized as major components of adolescents' quality of life since teens are changing their social role and desire to be socially accepted by their peers, school and community [35]. In particular, during adolescence there is a reorganization of the relationship with parents, thus this is a very important issue to be addresses. Finally, VSP-A focuses on the well-being, feelings and perceptions of the teens, while PedsQL and CHQ focus on functioning (what children or adolescents can do). Undoubtedly, functional status affects one's quality of life, but other aspects such as relationships, life satisfaction and well being should be assessed [36]. From the adults' perspective, quality of life and health status are considered different constructs and this distinction has to be considered when selecting instruments to be used in quality of life research [37].

When our study was implemented, the parent's form of the CHQ (CHQ-PF50) was the only generic tool validated in Brazil. Thus, a validated version of the VSP-A might fill a gap in the instruments available to measure HRQoL in Brazil and might be useful to complement the assessment of Brazilian adolescents' health. Nevertheless, when a HRQoL measure is to be employed across cultures and meaningful cross-cultural comparisons are expected, the tool needs to show not only its reliability and validity in each cultural context but also the equivalence between the different versions of the measure [38].

The purpose of this study was to cross-culturally adapt and validate the Brazilian-Portuguese version of the VSP-A for use in healthy adolescents.

## Methods

### Study population and design

The sample consisted of adolescents attending two public high schools in São Gonçalo City, located 30 km from the state capital of Rio de Janeiro and comprising a population of 960 631 inhabitants, of which 16% are adolescents. Of the 147 319 children and adolescents attending private or public schools, 77% attend public schools [2]. The socioeconomic position of the pupils' families of the targeted schools is low and similar to that of the population of adolescents attending public schools in São Gonçalo City. All adolescents aged 14 to 18 years from both schools were invited to participate.

The students were proposed to complete the HRQoL questionnaire as well as a symptom checklist, and provided socio-demographic information (age, gender and the main occupation of the head of the household). The socio-economic position was derived from the occupation of the head of the household. The jobs or occupations were classified into two groups: elementary and professional. Elementary are distinguished from professional occupations, which require graduate-level education and are associated with higher socio-economic positions. Students were asked whether they would agree to be interviewed again at about a two-week interval.

The Ethics Committee of Rio de Janeiro State University approved the study, and all adolescents signed an informed consent form. Informed parental consent was not required since the adolescents, at least 14 years old, were considered to be legally able to independently consent to participate in research evaluating their own quality of life.

### HRQoL Measure

The HRQoL measure to be adapted and validated in Brazilian Portuguese was the VSP-A, a self-reported, easy-to-complete, reliable and valid questionnaire for ill and healthy adolescents [28,29]. It comprises 36 questions divided into ten scales. The adolescents indicated on a 5-point scale the frequency or intensity of each item in the last four weeks. Negatively worded items were reversed so that higher scores indicated higher HRQoL levels. A score for each dimension scale was calculated as well as a total index (VSP-A index), derived by summing the scales, that can be interpreted as a global rating of the overall adolescent's HRQoL. Scores were linearly transformed to a 0-100 scale with 100 indicating the highest HRQoL.

### Symptoms measure

The adolescents completed the Psychosomatic Symptom Checklist (PSSC), a self-administered questionnaire composed of 17 items comprising common physical and psychological symptoms (e.g., headache, fatigue and depression) previously adapted for use in Brazil [39]. They recorded, on a 5-point scale, the frequency of each symptom in the last year. Higher scores determined higher frequency. Adolescents were considered symptomatic if the reported symptoms occurred at least once a week and asymptomatic if symptoms were reported less than once a week.

### Cross-cultural adaptation and validation process

The first step of the process consisted in producing a Brazilian version of the VSPA, showing semantic equivalence with the original French version. Second, the psychometric properties of the Brazilian version were investigated to check whether this version behaves similarly to the original French version, considering not only the reliability and internal construct validity of the VSPA, but also differential item functioning and external validity studies.

#### Brazilian translation and conceptual equivalence

**Figure 1:**
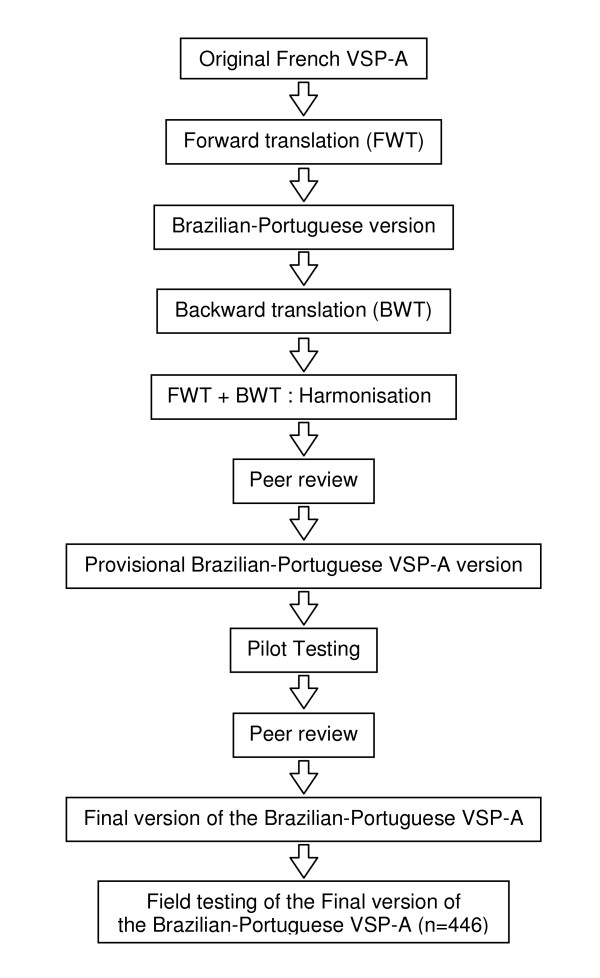
**Procedure for the cross-cultural adaptation of the VSP-A**.

A forward-backward translation was performed. The French version was translated into Brazilian-Portuguese by a Brazilian-Portuguese native speaker with a high level of fluency in Portuguese and French. The back-translation into French was undertaken independently of the forward-translation by a French native speaker. A panel of specialists, pediatricians and researchers experienced in the cross-cultural adaptation of instruments, fluent in French, discussed the divergences observed between the back-translation and the original French version in order to identify the difficulties emerged from the translation process. These difficulties were discussed with the authors of the original instrument and the items were reworded where agreement could not be reached. Then, a provisional VSP-A version was established and pilot-tested in a public high school in the municipality of São Gonçalo, in a sample of 14 adolescents (age range 14-16 years, 64.2% girls) that were not included in the validation study sample. All the students in the pilot test signed an informed consent form. They were asked whether the items were clear and understandable, and to rate the level of difficulty and relevance of each item. They were also asked to make suggestions in order to modify items they found difficult or irrelevant. At the end of the questionnaire there was an open question asking the respondents to report their opinions, suggestions or comments on the instrument. The adolescents all agreed that the Brazilian-Portuguese version of the VSP-A was clear, that the language was of common use, that the items were relevant and the instrument was in a comprehensive format. The results of the pilot-test were peer-reviewed yielding a final version of the Brazilian-Portuguese VSP-A conceptually equivalent to the French original VSPA and linguistically appropriate for use among Brazilian adolescents. This final version of the Brazilian-Portuguese VSP-A was implemented in a validation field study conducted among a large sample of students (Figure 1).

Internal construct validity. Internal construct validity was assessed through confirmatory factor analysis (CFA), using the French VSP-A scales as a reference. We aimed to determine how well the model generated from the results of the original VSP-A fit the data obtained in Brazil [40]. Maximum likelihood CFA using polychoric covariance was used to test the fit of these ordinal data to the model. The adequacy of the model was analysed using a global index that was responsive to sample size and complexity of the model (root mean square error of approximation - RMSEA), as well as an incremental index that was less dependent on the sample size (comparative fit index - CFI). An RMSEA lower than 0.05 indicated a good fit while values lower than 0.08 indicated a fair fit. CFI was expected to be above 0.9 for the model to adequately fit the data.

The structuring of items into scales was also investigated through multi-trait-multi-item analysis. Item-internal consistency (IIC) was assessed by correlating each item with its scale. IIC was supported when an item-scale correlation was 0.4 or greater. Item-discriminant validity (IDV) was assessed by determining the extent to which an item correlated significantly higher with its own scale (corrected for overlap) than with other scales ("scaling successes"). Multi-trait scaling analyses were summarised using tests of individual item scaling success and calculating the percentage of item scaling successes relative to the total number of items.

Correlations between scales of the VSP-A Brazilian-Portuguese version were also examined. The VSP-A domains were expected not to be highly correlated, giving evidence that the different scales measured different approaches of the same concept: HRQL.

#### Unidimensionality of each scale was assessed using Rasch analysis

Scalability was assessed using item goodness-of-fit statistics (INFIT); INFIT between 0.7 and 1.2 indicated that the scale's items tended to measure the same concept [41].

#### Reliability

Internal consistency and test-retest reliability were determined. Cronbach's alpha coefficient was calculated to assess the internal consistency of the scales. Estimates greater than 0.7 were sought [42].

#### Reproducibility

The reproducibility of the Brazilian version was evaluated based on a test-retest procedure calculating the intra-class correlation coefficient (ICC) among a sample of 195 adolescents who were retested about two weeks later (± one week) and did not report any major life events, namely disorders that led to absenteeism or hospitalization, in this time interval.

#### Differential item functioning (DIF)

Cross-cultural equivalence of the VSP-A items was evaluated by investigating whether the translated Brazilian items function the same way as the original French items. We compared the INFIT of Brazilian and French samples to identify invariance of item calibrations. DIF analyses were performed to describe the performance of items and dimensions across different groups and to test their cross-cultural applicability [43]. If an item functions differently in the original and translated versions, it exhibits DIF. Non-uniform DIF, which exists when the probability of giving a particular answer at a given level of health varies both by country and levels of health, was calculated. Zumbo's ordinal logistic regression analysis was used to detect DIF [43].This approach enabled quantification of the magnitude of DIF by a pseudo-R² difference (Δ-R²) measure, expressing the increase in explained item variance by including the variable for group membership. A cut-off point of 2% was used for DIF [43].

#### Acceptability

The percentage of respondents achieving the lowest (floor effect) and the highest (ceiling effect) score in each dimension and the percentage of missing dimension scores were calculated. Floor and ceiling effects exceeding 15% were considered high [44]. A large amount of missing scores as well as high floor or ceiling effects would be indicative of difficulty in using the questionnaire and interpreting scores (reflecting acceptability for the clinicians, or "users"). The mean time needed to complete the VSP-A Brazilian-Portuguese version was also calculated as the difference between start time, namely the time the questionnaire was given to the students, and the end time, when they returned the instrument fulfilled. A short time of completion suggests a greater acceptability for both "users" and respondents.

#### External construct validity

Based on the literature, some hypotheses were generated in order to investigate the external construct validity of the VSP-A Brazilian-Portuguese version.

First, we hypothesised that, within the Brazilian sample, dimension scores would vary between subgroups of adolescents according to gender and the symptoms reported. We expected HRQoL scores to be lower in symptomatic adolescents, and Brazilian girls to score lower in dimensions such as physical and psychological well-being and body image [45-47].

#### Second, we also compared adolescent HRQoL between French and Brazilian samples

The Brazilian sample belongs to a low socioeconomic class and faces problems such as violence and teenage pregnancy, so we expected these adolescents to score lower in the physical and psychological well-being and sentimental and sexual life scales when compared to French teens [48-51]. We also expected Brazilian adolescents to score lower in the school performance scale when compared to French adolescents [52].

One-way analysis of variance (ANOVA), Mann-Whitney tests and the multivariate general linear model were used to compare the dimension scores between: (1) boys and girls; (2) symptomatic and non-symptomatic adolescents; and (3) Brazilian and French pupils.

Description of the population features. Standard descriptive statistics of the sample characteristics were computed: means and standard deviations for continuous variables, and effectives and percentages for categorical variables. Groups of adolescents were compared using Chi^2 ^tests or accurate Fisher tests for qualitative variables, and ANOVA or Mann-Whitney tests for quantitative variables depending on the conditions of application. For all tests, statistical significance was set at p < 0.05.

SPSS 13.0, MAP - R (Multi-trait analysis program), LISREL 8.52 and Winsteps 3.42 software were used.

## Results

### Sample Characteristics

A total of 446 adolescents completed the Brazilian-Portuguese version of the VSP-A (response rate 87.9%). The ages ranged between 14 - 18 years (mean 16.6 years, SD 1.1); 53.6% were girls. Of the 373 students (83.6% of the respondents) that reported the occupation of the head of the household, 85.7% were elementary service workers, 7.2% professional workers, 3.6% housewives, 2.9% retired and 0.6% unemployed. The pupils who were not included did not differ significantly from those included with regard to demographic features. Half of the 396 Brazilian adolescents (88.8% of the respondents) answering the Psychosomatic Symptom Checklist reported at least one symptom per week. Girls tended to report significantly more symptoms when compared to boys (Table 1).

**Table 1:** Percentage and number of Brazilian boys and girls reporting psychosomatic symptoms at least once a week in the last year (n = 396 respondents on the PSSC).

Symptoms	Total (n = 396)		Boys (n = 172)		Girls (n = 224)	
	**N**	**%**	**N**	**%**	**N**	**%**

Headache *	59	14.9	8	4.7	51	22.8

Fatigue *	72	18.2	24	14.0	48	21.4

Dizziness *	48	12.1	10	5.8	38	17.0

Weakness *	34	8.6	4	2.3	30	13.4

Abdominal pain *	30	7.6	4	2.3	26	11.6

Insomnia	35	8.8	11	6.4	24	10.7

Depression *	19	4.8	1	0.6	18	8.0

Constipation *	21	5.3	1	0.6	20	8.9

Nausea	14	3.5	3	1.7	11	4.9

At least one symptom in the last year*	199	50.3	62	36.0	137	61.2

Two or more symptoms in the last year*	119	30.1	30	17.4	89	39.7

* Differences between boys and girls significant at p < 0.05 (chi-square test).

### Internal Construct Validity

The CFA showed an acceptable fit (RMSEA = 0.054 and CFI = 0.93). Examining IIC and IDV, only item 28 ("Have you been in good physical shape?") was more highly correlated with the vitality scale (r = 0.41, p < 0.05) than with the physical well-being scale (r = 0.28, p < 0.05), which is its own scale (Table 2). Ninety-four percent of item-hypothesised scale correlations were greater than 0.4 and 98% of item-hypothesised scale correlations were greater than the correlation of the item to the other scales, demonstrating scaling success. The patterns of item-dimension scales correlations support both IIC and IDV. As expected, VSP-A dimension scores were at most moderately correlated with each other; the highest correlations were found for the following pairs: school performance - relationship with teachers (r = 0.44; p < 0.01), vitality - physical and psychological well-being (r = 0.41 and r = 0.45, respectively; p < 0.01), psychological well being - physical well-being (r = 0.49; p < 0.01).

**Table 2:** Descriptive statistics of the Brazilian-Portuguese version of the VSP-A (N = 446).

Scale	Missing data (%)	Ceiling - Floor effect (%)	IIC	IDV	ICC (N)
PsyWB	5 (1.1%)	5.8 - 0.4	0.47-0.61	-0.09-0.43	0.55* (193)

PhyWB	1 (0.2%)	3.1 - 0.2	0.28-0.51	-0.02-0.41	0.58* (193)

VIT	1 (0.2%)	6.1 - 0.7	0.65-0.73	0.20-0.44	0.73* (193)

RT	4 (1.1%)	1.3 - 0.4	0.53-0.69	0.02-0.37	0.69* (191)

SP	7 (1.6%)	6.5 - 5.4	0.73-0.73	-0.02-0.42	0.70* (189)

RF	0	3.8 - 0.2	0.43-0.64	0.01-0.39	0.82* (195)

RP	0	4.9 - 4.3	0.51-0.65	0.00-0.43	0.83* (193)

BI	19 (4.3%)	15.9 - 6.1	0.46-0.46	-0.07-0.36	0.55* (193)

LA	0	1.3 - 0.7	0.46-0.52	-0.02-0.38	0.75* (193)

SSL	133 (42%)	13.9 - 7.0	0.63-0.63	0.01-0.34	0.74* (120)

VSP-A dimensions: PsyWB - psychological well-being; PhyWB - physical well-being; VIT - vitality; RT - relationship with teachers; SP - school performance; RF - relationship with friends; RP - relationship with parents; BI - body image; LA - leisure activities; SSL - sentimental and sexual life; VSP-A scores ranging from 0 (lowest quality of life) to 100 (highest quality of life). Floor - % of cases achieving the lowest score; Ceiling - % of cases achieving the highest score; IIC- Item internal consistency; IDV - Item-discriminant validity : minimum - maximum correlation coefficient; ICC - Intra-class test-retest correlation coefficient. * p < 0.001

### Unidimensionality

The overall scalability of the VSP-A in Brazil was satisfactory. Most of the items showed a good fit to the Rasch model (INFIT ranging between 0.7-1.2) with the exception of the items "Have you been anxious, worried?" in the psychological well-being dimension (INFIT = 1.3) and "Have you been accepted, respected by your teachers?" in the relationship with teachers dimension (INFIT = 1.3).

### Reliability

Cronbach's alpha coefficients ranged from 0.60 to 0.85 (Table 2). The VSP-A index Cronbach's alpha was 0.87.

In total, 195 (43.7% of the initial sample) adolescents participated in the retest (Table 2). This subsample did not differ from that not included in the retest in terms of socio-demographic characteristics or scores on the various VSP-A scales at baseline. The dimension scores showed fair to good reproducibility (ICC ranging from 0.55 to 0.85).

### Cross-cultural item functioning

In general, the amount of DIF between the samples of French and Brazilian adolescents was low; only 4 out of the 36 items showed significant DIF: "Have you had confidence in yourself, been sure of yourself?" (vitality scale), "Have you been able to get together with your friends?" (leisure activities scale), "Have you been in good physical shape?" (physical well-being scale) and "Have you been anxious" (psychological well-being). One item, "Have you been discouraged'', was at the upper threshold for DIF (Table 3).

**Table 3:** Internal consistency (Cronbach's alpha coefficient), Unidimensionality (INFIT) and Differential Item Functioning (DIF) analyses of the 10 dimensions of VSPA between the Brazilian and the French adolescents

Dimension/Items	INFIT Brazil	INFIT France	DIF - Non-uniform
**Vitality**			

15 - Happy	1.1	1.1	0,8

31 -Good mood	0.9	1.0	1,1

32- Bright side life	0.8	0.8	0,2

33- Fine round you	0.9	0.9	0,9

34 - Confidence in yourself	1.2	1.2	**4,6**

*Cronbach's alpha*	*0.85*	*0.84*	

**Relationship with teachers**			
22- Helped teachers	0.8	0.9	0,7

23- Understood teachers	0.9	0.8	0,3

24 - Accepted teachers	1.3	1.2	0,0

*Cronbach's alpha*	*0.79*	*0.77*	

**School performance**			
21- Happy with School grades	1.0	1.0	0,0

35- Good School results	1.0	1.0	0,1

*Cronbach's alpha*	*0.84*	*0.83*	

**Relationship with parents**			
6 - Tell Problems to parents	0.9	0.7	0,1

7 - Talk to parents	1.2	1.1	0,1

20 - Understood by parents	0.9	1.1	**2,0**

30- Parents give you advise	1.0	1.2	1,7

*Cronbach's alpha*	*0.80*	*0.80*	

**Relationship with friends**			
3 - Talk to your friends	1.0	0.9	0,8

4 - Confide problems to friends	1.0	1.2	1,9

5 - Express to friends	1.2	1.1	0,1

16 - Helped by friends	0.8	0.9	0,1

17 - Understood by friends	0.9	0.8	0,2

*Cronbach's alpha*	*0.77*	*0.78*	

**Leisure activities**			
1- Meet your friends	0.9	0.8	**3,1**

2 - Go downtown with your friends	0.9	1.0	1,3

8 - Invited home by your friends	1.0	1.0	1,1

9- Play outside	1.1	**1.2**	0,2

*Cronbach's alpha*	*0.71*	*0.81*	

**Body image**			

25 - Unsatisfied with your apparence	1.0	1.0	0,7

26 - Fat/thin/short/tall	1.0	1.0	0,5

*Cronbach's alpha*	*0.64*	*0.87*	

**Psychological well-being**			
10 - Preoccupied	0.8	0.7	0,2

11- Sad	1.0	0.9	0,1

12 - Stressed	1.2	1.1	0,0

13 - Discouraged	0.7	1.1	**2,1**

14 - Anxious	1.3	**1.2**	**2,7**

*Cronbach's alpha*	*0.76*	*0.85*	

**Physical well-being**			
27 - No energy	0.9	0.8	0,8

28 - Good physical shape	1.2	1.2	**6,8**

29 - Weak or tired	0.8	0.8	1,1

36 - Pain	1.0	1.1	0,6

*Cronbach's alpha*	*0.60*	*0.72*	

**Sexual and sentimental life**			
18 - Sentimental Life	0.9	0.8	0,3

19 - Sexual Life	1.1	1.2	0,3

*Cronbach's alpha*	*0.70*	*0.72*	

INFIT: item goodness-of-fit statistic according to Rasch analyses, in bold values >=1.2

Non uniform DIF Zumbo's ordinal logistic regression analyses results, in bold: values >2.0%

### Acceptability

The mean completion time was 15 minutes. The amount of scale-level missing data was lower than 5%, except for the scale on sexual and sentimental life (42%). A ceiling effect was observed in the body image scale. In the other dimension scales, ceiling and floor effects were lower than 15% (Table 2).

### External Construct Validity

On the whole sample, the lowest mean score was found in the dimension of school performance, whereas the highest was in the dimension of psychological well-being; the total VSP-A index score was 58.6 ± 13.0 (Table 4). As expected, adolescents reporting at least one symptom per week presented significantly lower scores in the six dimension scales and the total score (Table 3). Supporting our hypotheses, girls scored significantly lower in five dimension scales and the total score (Table 3). Adolescents presenting with headaches also had lower scores in psychological well-being (72.3 ± 20.6 vs. 57.2 ± 21.4; p < 0.001), physical well-being (66.5 ± 17.1 vs. 50.8 ± 16.3; p < 0.001) and vitality scales (68.6 ± 21.4 vs. 51.8 ± 14.3; p < 0.001). Adolescents reporting depression scored lower in physical well-being (65.5 ± 17.6 vs. 39.8 ± 23.4; p < 0.001), psychological well-being (71.3 ± 19.7 vs. 34.6 ± 22.4; p < 0.001) and body image scales (63.3 ± 26.8 vs. 44.5 ± 39.8; p < 0.001). Fatigued adolescents scored lower in the following scales: vitality (68.8 ± 21.3 vs. 57.6 ± 21.8; p < 0.001), leisure activities (54.5 ± 20.8 vs. 47.6 ± 22.7; p < 0.001), physical well-being (67.6 ± 17.9 vs. 50.6 ± 16.2; p < 0.001) and psychological well-being (72.6 ± 20.4 vs. 57.3 ± 21.2; p < 0.001).

**Table 4:** Mean and standard deviation of the scores of the Brazilian-Portuguese VSP-A scales according to the gender and presence of psychosomatic symptoms reported on the PPSC.

Scale	Whole sample	Gender			Presence of symptoms		
	N = 446	Boys N = 207	Girls N = 239	p-value (ANOVA)	No symptoms N = 197	At least one symptom once a week N = 199	p-value (ANOVA)

PsyWB	69.4 ± 20.6	75.4 ± 18.6	64.2 ± 0.7	p < 0.001	76.5 ± 18.1	62.8 ± 21.3	p < 0.001

PhyWB	64.4 ± 18.7	69.4 ± 17.9	60.0 ± 8.3	p < 0.001	72.0 ± 15.1	56.1 ± 18.2	p < 0.001

VIT	66.3 ± 22.2	70.1 ± 21.0	62.9 ± 22.5	p < 0.001	71.5 ± 20	61.4 ± 22.9	p < 0.001

RT	49.4 ± 24.4	48.9 ± 23.3	49.9 ± 25.2	0.68	51.9 ± 2.3	47.2 ± 26.0	p < 0.05

SP	48.8 ± 26.2	48.3 ± 26.4	49.1 ± 25.9	0.75	51.6 ± 24.5	47.6 ± 27.1	0.12

RF	61.4 ± 20.7	54.9 ± 18.8	67.0 ± 20.8	p < 0.001	61.2 ± 20.7	62.8 ± 20.9	0.4

RP	53.0 ± 26.2	55.4 ± 24.9	51.0 ± 27.2	0.80	55.4 ± 25.1	50.7 ± 27.4	0.8

BI	61.6 ± 28.1	65.0 ± 25.2	58.8 ±30.0	0.023	66.2 ± 26.9	59.2 ± 27.8	p < 0.05

LA	53.6 ± 20.8	57.7 ± 19.5	50.0 ± 1.3	p < 0.001	56.1 ± 19.7	50.3 ± 21.9	p < 0.001

SSL	59.6 ± 32.4	59.3 ± 30.7	59.9 ± 4.2	0.87	57.7 ± 32.1	61.8 ± 33.1	0.26

Index	58.6 ± 13.0	60.7 ± 12.1	56.3 ± 3.5	p < 0.01	62.3 ± 11.7	55.3 ± 13.5	p < 0.001

VSP-A dimensions: PsyWB - psychological well-being; PhyWB - physical well-being; VIT - vitality; RT - relationship with teachers; SP - school performance; RF - relationship with friends; RP - relationship with parents; BI - body image; LA - leisure activities; SSL - sentimental and sexual life; VSP-A scores ranging from 0 (lowest quality of life) to 100 (highest quality of life).

Mean score comparisons adjusted for age and gender showed that Brazilian teens scored significantly lower in sexual and sentimental life (French adolescents mean score 65.9 ± 31.7; p < 0.001), leisure activities (French mean score 64.8 ± 26.2; p < 0.001) and relationships with friends (French mean score 65.6 ± 23.0; p < 0.001) when compared to French adolescents. On the other hand, Brazilian adolescents scored significantly higher in psychological well-being (French mean score 63.3 ± 24.4, p < 0.001), vitality (French mean score 61.5 ± 22.7; p < 0.001) and relationships with teachers (French mean score 41.9 ± 26.0, p < 0.001). No significant differences were found, although Brazilian ratings tended to be lower than French ones in physical well-being (French mean score 62.2 ± 21.2, p= 0.94), school performance (French mean score 51.5 ± 24.6, p= 0.51), and body image (French mean score 63.2 ± 34.3, p= 0.94).

## Discussion

The objective of this study was to culturally adapt and validate the Brazilian version of the VSP-A (Table 5). The choice of this instrument was motivated by the fact it was a generic instrument specifically developed to assess adolescent HRQoL taking care the adolescents' perceptions be reflected by following an extensive qualitative approach combining individual interviews and focus groups in both healthy adolescents and adolescents with a large variety of health conditions [28,29]. Moreover, the VSP-A was developed to be administered in a form of a questionnaire self-reported by the adolescents. At the time of the study implementation, no other generic HRQoL questionnaire validated in Brazilian fulfilled all these requirements. The CHQ-PF50 (parent form) [18,20] could have been added as a concurrent measure to explore the convergent validity of the instruments. However, this would have well complicated the study design since parents' reports should have been collected in addition to adolescents' ones and was likely to jeopardize the study carrying-out. Because the main objective of this study was to address the adolescents self-reports of HRQoL we chose not to collect parents' reports neither with the VSP-A parent version nor with the CHQ-PF50. More recently, the PedsQL was adapted and tested with Brazilian adolescents and their parents [25-27]. The comparison of these different HRQoL questionnaires in a large sample of Brazilian-Portuguese adolescents would bring useful information for the users of HRQoL helping in the choice of the questionnaires the most suitable to the study purpose.

**Table 5:** Brazilian-Portuguese version of the VSP-A.

	Item meaning	Brazilian-Portuguese Version
	During the past 4 weeks,	Nas últimas 4 semanas,

1.	Have you been able to get together your friends?	Você pôde encontrar em grupo com seus colegas, amigos ou amigas?

2.	Have you been able to go out (to the mall/shopping centre, to a movie/cinema...)?	Você pôde sair (passear na rua, ir ao shopping, ir à piscina, praia ou ao cinema)?

3.	Have you been able to talk with your friends ?	Você pôde conversar com seus colegas, seus amigos ou suas amigas?

4.	Have you been able to confide in, talk about your problems with your friends?	Você pôde abrir-se, falar de seus problemas com seus colegas, amigos, suas amigas?

5.	Have you been able to express yourself freely to your friends?	Você pôde expressar-se livremente, dar sua opinião a seus colegas, amigos ou amigas?

6.	Have you been able to confide in, talk about your problems with your parents?	Você pôde abrir-se, falar de seus problemas com seus pais?

7.	Have you been able to express yourself freely to your parents?	Você pôde expressar-se livremente, dar sua opinião a seus pais?

8.	Have you been invited your friends' home or invited them to yours?	Você foi para a casa de seus colegas, amigos/suas amigas?

9.	Have you played outside with your friends (catch, bicycling, roller-blading...)?	Você saiu ao ar livre para se divertir com seus colegas, amigos e amigas (passear, andar de bicicleta, jogar vôlei ou futebol)?

10.	Have you been anxious, worried?	Você se sentiu inquieto/a, preocupado/a?

11.	Have you been sad, depressed?	Você se sentiu triste, deprimido/a?

12.	Have you been stressed, nervous, irritable?	Você se sentiu estressado/a?

13.	Have you been easily discouraged?	Você se sentiu facilmente desanimado/a?

14.	Have you been anxious, worried about the future?	Você se sentiu angustiado/a, ou com medo ao pensar no futuro?

15.	Have you been satisfied with your life?	Você se sentiu contente, satisfeito/a com sua vida?

16.	Have you been helped by your friends when you needed it?	Você se sentiu apoiado/a, ajudado/a por seus colegas, seus amigos ou suas amigas?

17.	Have you been understood, reassured by your friends?	Você se sentiu compreendido/a, tranqüilizado/a por seus colegas, seus amigos ou suas amigas?

18.	Have you been satisfied with your romantic relationship with your girl/boy-friend?	Você se sentiu satisfeito/a em sua vida sentimental com seu namorado/sua namorada?

19.	Have you been satisfied with your sex life?	Você se sentiu satisfeito/a com sua vida sexual?

20.	Have you been understood, reassured by your parents?	Você se sentiu compreendido/a, tranqüilizado/a por seus pais?

21.	Have you been satisfied with your school grades?	Você esteve satisfeito/a com suas notas na escola?

22.	Have you been helped by your teachers?	Você foi ajudado/a por seus professores?

23.	Have you been understood by your teachers?	Você se sentiu compreendido/a por seus professores?

24.	Have you been accepted, respected by your teachers?	Você se sentiu aceito/a, respeitado/a por seus professores?

25.	Have you been feeling overly conscious of your body, physical appearance?	Você se sentiu complexado/a com seu físico, sua aparência?

26.	Have you been feeling too fat or too skinny, too tall or too small?	Você se sentiu gordo/a ou magro/a demais, alto/a ou baixo/a demais?

27.	Have you had little energy?	Você se sentiu sem energia?

28.	Have you been in good physical shape?	Você se sentiu em boa forma física?

29.	Have you been feeling weak, tired?	Você se sentiu fraco/a, cansado/a?

30.	Have your parents given you advice?	Seus pais lhe deram conselhos?

31.	Have you been in good spirits?	Você sentiu que estava animado/a?

32.	Have you been looking on the bright side of things?	Você sentiu que estava disposto a ver o lado bom da vida?

33.	Have you been feeling that all around you was going well?	Você sentiu que tudo ia bem à sua volta?

34.	Have you had confidence in yourself, been sure of yourself?	Você sentiu confiança em si mesmo/a?

35.	Have you been getting good grades in school?	Você sentiu que teve bons resultados na escola?

36.	Have you had aches and pains?	Você sentiu dores, mal estar em alguma parte do corpo?

Besides, although the benefits of collecting adolescent's self-reports of HRQoL are widely stressed, the fundamental role for parent proxy-reports in clinical trials and health services research should be mentioned. In situations where the adolescent is unable to complete a HRQoL tool, such as in cognitive deficiencies or severe diseases, HRQoL assessment should rely on proxy reports or else be given up. Furthermore, given that perceptions of the parents or guardians often drive health care utilization, parent proxy-reports may be useful to better understand factors impacting the access to healthcare dedicated to children and adolescents. Parent-proxy reports can provide complementary information regarding adolescents' mental health and well-being [53,54]. Therefore further studies are necessary to evaluate the applicability and validity of the parent-proxy version of the VSP-A in the Brazilian context [55].

Cross-cultural adaptation and validation of a HRQoL instrument requires that the translated tool not only be linguistically appropriate for use in the target population and conceptually equivalent to the original one, but also show satisfactory psychometric properties and cross cultural measurement equivalence. One interest of this study was to assess the validity of the Brazilian version of the VSP-A using methods relying on both classical test theory and item response theory. Zumbo's method using ordinal logistic regression (OLR) was favoured since OLR-based techniques were found to be superior to Mantel-Haenszel in identifying items that had nonuniform DIF [56].

The results support the structural validity of the instrument in the Brazilian population. The domains described in this French adolescent measure were found to be appropriate to investigate HRQoL in Brazilian pupils, since the results of CFA and Multi-trait-multi-item analysis confirmed its multidimensional structure was satisfactory with one limitation regarding item 28 ("Have you been in good physical shape?"). This item originally belonging to the physical well-being scale showed a significantly higher correlation with the vitality scale. Regarding unidimensionality, item misfit was observed in 2 of 36 items, but these items showed neither DIF nor scalability problems. However, the item 28 exhibited values at the upper threshold for misfit according to Rasch analysis and presented DIF. Internal consistency reliability remained satisfactory despite of slightly lower Cronbach's alphas found for the VSP-A index, and for body image and physical well-being scales, compared to those obtained in the original study (0.87 *vs. *0.91, 0.64 *vs. *0.85 and 0.60 *vs. *0.84, respectively) [28]. Regarding item functioning across cultures, most VSP-A items appeared to function equivalently across France and Brazil. Although 4 of 36 items showed significant DIF (11%), the effect sizes for the DIF were small for all the items except for two of them (28 "good physical shape" and 34 "confidence in yourself") [43]. Since it is the first psychometric analysis of the Brazilian VSPA, these results should be verified when the scale will be used in distinct samples. The translation of these items should also be verified. Nevertheless, when DIF is detected for an item, several methods have been proposed for correcting for DIF: one of them is based on the calibration of item parameters using Item Response Theory analysis in order to allow the calculation of person Rasch scores adjusted for DIF [57]. At last, items exhibiting DIF would be candidate for deletion when a shorter version of the questionnaire will be elaborated.

Thus, the major concern was about item 28, "good physical shape", since the various analyses showed that it functioned differently in the Brazilian version compared to the French original questionnaire. This fact will lead us to review carefully the translation process of this item, but also to investigate more in-depth the relevance or specificities of this concept in the Brazilian adolescent population.

With regard to the reproducibility testing, the test-retest method with students agreed to be interviewed twice may have led to several bias. One the one hand, there is a potential for learning, carry-over, or recall effects. A very short time interval makes the carryover effects due to memory, practice, or mood more likely, whereas a longer interval increases the chances that a change in status could occur. The two-week time interval chosen in our study is generally believed to be a reasonable compromise between recollection bias and unwanted (on the part of the investigator) change in health condition [58]. To reduce the potential change in health condition, the adolescents were asked whether major life events leading to either hospitalization or absenteeism occurred in the given time period. On the other hand, although less than half of the whole sample was involved in the retest, the existence of a selection bias is not likely since no difference was found between adolescents included in the retest and those who were not with regard to major features. Overall, the potential for bias in the test-retest data remains limited.

External construct validity of the VSP-A was supported in this Brazilian sample. The hypotheses that symptomatic adolescents would score lower than non-symptomatic adolescents and that girls would score lower on the body image, physical and psychological well-being scales were supported. Concerning gender, the results were similar to those found when the VSP-A was applied to French and Spanish samples; girls also scored lower in the vitality and body image scales, and higher in the relationship with friends scale [28-31]. In general, girls tend to be more dissatisfied with their body weight than boys [45-47]. These results are in line with findings of other studies demonstrating that girls in general have lower scores on the HRQoL when compared to boys [46].

Regarding the Brazilian adolescent population, a study found lower socioeconomic levels and lower maternal literacy were associated with poor psychological well-being [49]. Brazilian researchers studying the prevalence of exposure to physical violence among adolescents attending public schools found that 25% of adolescents witnessed someone being shot and 14% witnessed someone being killed [50]. These findings led us to hypothesise that Brazilian adolescents would score lower in the psychological well-being scale. Contrary to our expectations, Brazilian adolescents scored higher on this scale when compared to French adolescents. One explanation could be that Brazilian adolescents tended to deny questions such as "Have you been sad, depressed?", "Have you been anxious, worried?" or "Have you been stressed, nervous, irritable?". However, this result might also be attributable to the fact that French adolescents tended to report low psychological well being scores. This latter hypothesis is supported by another study where French adolescents tended to present lower psychological well-being scores when compared to adolescents from other European countries [48].

Approximately 40% of Brazilian adolescents (16-19 years) reported sexual initiation before age 15 [51]. Research developed in three Brazilian state capitals found that almost 30% of girls reported pregnancy during adolescence (70% of which were unwanted) and demonstrated an inverse relationship between educational level and rate of pregnancy [59]. Teenage pregnancy is a problem that accompanies the initiation of sexual activity at increasingly younger ages. It is of particular concern because of its social, economical and educational impact on adolescent HRQoL [59,60]. This led us to hypothesise Brazilian adolescents would score lower in the sexual and sentimental life dimensions, which was confirmed.

Poor educational performance in Brazil is mainly due to the inadequate quality of public schools; problems include low salaries for teachers and shortages of textbooks and instructional materials, as well as ill-equipped school buildings [52]. As this study was conducted in a public school setting, and considering that an unsatisfactory educational system plays an important role in academic achievement, we expected Brazilian adolescents to score lower in the school performance scale. However, a statistically significant difference was not obtained in this scale or in the overall VSP-A index.

Apart from the dimension of sentimental and sexual life, the low level of missing data confirmed the instrument acceptability. The pupils were oriented to skip any item they chose not to answer. Possibly, some did not feel comfortable answering both items about sexual and sentimental life, but the score of this dimension was considered as missing when at least one item was not answered. Nevertheless, answering only one of the items would remain meaningful. The same degree of missing scores for this scale was observed in the French sample. There was no gender difference in the completion rate of those items. To avoid this issue, the scoring method could be reviewed so that score could be computed as for the other dimensions when at least half of the items are answered, and thus as soon as one of the items of the sentimental and sexual life dimension is answered.

At last, the high frequency of psychosomatic symptoms reported by the students (88.8% of the sample with at least one symptom) could be surprising. Nevertheless, the symptoms assessed in the Psychosomatic Symptom Checklist represent very common complaints, such as headache, backaches, insomnia, fatigue, weakness, constipation, diarrhea, eye pain associated with reading, nausea and stomach pain. As the adolescents were in a particular moment of their lives, because they were finishing the high school and preparing to enter a college or job market, they could present a variety of symptoms. One explanation could be that the exams required to enter a college influence the somatization in this community sample.

Some limitations of this study have to be addressed. First, we did not perform an extensive testing of the instrument through focus groups and individual interviews with Brazilian adolescents, in order to better evaluate conceptual equivalence and content validity of the instrument. Nevertheless, some interviews with adolescents were conducted during the adaptation process (pretesting including cognitive debriefing with 14 adolescents), and during the validation study (debriefing interviews after completion of the questionnaire for part of the sample). Pupils were also encouraged to note topics or questions that they considered irrelevant or conversely missed at the end of the questionnaires. Apart from issues relating to item 28 that should be further investigated, the use of the questionnaire in almost 500 adolescents showed no major issues and supports the appropriateness of the scales in the Brazilian socio-cultural context. Second, the recruitment of students from low-income neighbourhoods in one city in southern Brazil could impede the generalisability of our sample. However, we found that 7.2% of the heads of households had a professional occupation, which is similar to the proportion reported for the Brazilian southern region overall (7.7% of the population has more than 10 years of study, indicating superior academic achievement) [61]. Another limitation of our study is that we included only adolescents attending school; those who have dropped out of school and would probably present a lower level of HRQoL were not enrolled in this study. Additional investigations will be necessary to evaluate how the quality of life of this group of adolescents might differ from our sample, although study design and implementation in this population will likely be challenging. Furthermore, the current data support the application of the Brazilian-Portuguese version of the VSP-A in healthy adolescents, in school settings. Further evaluation of the instrument is needed, including analysis of item performance and scale validity among adolescents with various diseases or health conditions. The next studies will contribute to further testing of the validity of the instrument in different contexts of use. The accumulation of results will also allow us to identify which findings are consistent across studies since all confounding factors cannot be controlled for in a single study. More specifically, sensitivity to changes needs to be investigated in order to use this scale for an evaluative purpose.

## Conclusions

Overall, the Brazilian version of the VSP-A seems to be a psychometrically acceptable instrument for measuring HRQoL in healthy Brazilian adolescents. It is a simple tool that is easy to administer and score. It provides valuable information on different dimensions of the adolescent HRQoL, and the total score represents a quantification of the general adolescent level of HRQoL. In that, it might serve as a starting point for more specific clinical investigations and interventions. A shorter version of the VSP-A would be suitable for use in our population and easier to use in day-to-day practice. A 12-item short version of the VSP-A is already available for use in France [62], and further work is needed to explore the psychometric properties of a shorter version in our context.

## Competing interests

The authors declare that they have no competing interests.

## Authors' contributions

MTA participated in the conception and design of the study, in the data collection, performed the statistical analyses and drafted the manuscript. PA coordinated the cross-cultural adaptation of the VSP-A, participated in the conception of the study, in the statistical analyses, in the interpretation of the data and revised the manuscript critically. SR participated in the statistical analyses and in data interpretation. GLW participated in the conception and design of the study, in the data collection and revised the manuscript critically. MCS coordinated the cross-cultural adaptation of the VSP-A, participated in the conception of the study, in the statistical analyses, in the interpretation of the data and revised the manuscript critically. All authors read and approved the final manuscript.

## Pre-publication history

The pre-publication history for this paper can be accessed here:

http://www.biomedcentral.com/1471-2431/11/8/prepub
